# Mortality Causes in Captive Cantabrian capercaillie (*Tetrao urogallus cantabricus*) in Spain

**DOI:** 10.3390/ani13071255

**Published:** 2023-04-05

**Authors:** Alberto García-Rodríguez, Gloria Herrero-García, María Gracia de Garnica García, Álvaro García Esgueva, Ramón Balsera, Álvaro Oleaga, Daniel Fernández, Javier Amado, Luis José Royo, María José García Iglesias, Ana Balseiro

**Affiliations:** 1 Departamento de Sanidad Animal, Facultad de Veterinaria, Universidad de León, 24071 León, Spain; albertogarciarodriguez1985@gmail.com (A.G.-R.); gherrg01@estudiantes.unileon.es (G.H.-G.); mgarng00@estudiantes.unileon.es (M.G.d.G.G.); agarce05@estudiantes.unileon.es (Á.G.E.); mjgari@unileon.es (M.J.G.I.); 2 Micros Veterinaria, S.L., C/Profesor Pedro Cármenes, Campus de Veganzana, 24007 León, Spain; 3 Consejería de Fomento, Ordenación del Territorio y Medio Ambiente, 33007 Oviedo, Spain; ramon.balserariesgo@asturias.org; 4 Sociedad de Servicios del Principado de Asturias S.A. (SERPA), La Laboral, 33203 Gijón, Spain; alvaroleaga@yahoo.es (Á.O.); danielfernandez.vet@gmail.com (D.F.); 5 Consejería de Medio Rural y Cohesión Territorial, 33299 Gijón, Spain; javier.amadofernandez2@asturias.org; 6 Departamento de Biología Funcional, Genética, Universidad de Oviedo, 33006 Oviedo, Spain; royoluis@uniovi.es

**Keywords:** Cantabrian capercaillie, *Tetrao urogallus cantabricus*, captivity, mortality causes, pathology, bacterial diseases, aspergillosis, myopathy, valgus leg deformity

## Abstract

**Simple Summary:**

Here, we describe the main mortality causes of 29 Cantabrian capercaillies bred in captivity, and discuss how this new knowledge may provide relevant information for reducing their mortality and a better maintenance of the species in captivity. Most of the animals that were less than 2 months old died due to infectious diseases (14/16, 87.5%), while stress-related processes were the main cause of death in animals more than 7 months old (7/13, 53.85%). We also report two free-ranging adult males that died due to exertional myopathy.

**Abstract:**

The Cantabrian capercaillie (*Tetrao urogallus cantabricus*) is one of the most severely threatened subspecies of capercaillie. Its current population range is restricted to a small area of the Cantabrian Mountains (northwestern Spain), with only around 200 individuals remaining. As part of the national strategy for the conservation of the subspecies, the Cantabrian capercaillie Captive Breeding Center of Sobrescobio opened in 2009. Here, we use the information provided by the necropsies performed in this facility on 29 individuals (11 males, 13 females and 5 undetermined; 16 chicks and 13 adults) in order to describe the main mortality causes of captive-bred Cantabrian capercaillies. After necropsy, tissue samples were taken for evaluation using standard methods in histology and microbiology. The majority of the captive animals (18/29, 62.07%) died due to infectious diseases, mainly due to *Escherichia coli*, *Clostridium perfringens*, or *Aspergillus fumigatus* infection. The remaining 11 animals died due to stress-related processes (i.e., rupture of the heart apex and cardiomyopathy or neurogenic shock) (8/29, 27.59%), duodenal obstruction and coelomitis (1/29, 3.45%), perforation of the proventriculus and heart with a briar branch (1/29, 3.45%) or euthanasia due to a valgus leg deformity that prevented proper animal welfare (1/29, 3.45%). Young animals (i.e., younger than 2 months) died mainly due to infectious diseases (14/16, 87.5%), while stress-related causes were responsible for most adult deaths (7/13, 53.85%). We additionally report that two free-ranging adult males died due to exertional myopathy. This study provides relevant information for reducing mortality in captive capercaillies and improving both living conditions in captivity and the adaptation of these animals to the wild.

## 1. Introduction

Strategies for biological conservation are usually grouped into two main categories. While in situ approaches focus on the preservation of ecosystems and viable populations of species in their natural areas, ex situ strategies aim at the conservation of components of biological diversity outside of their natural habitats, usually having the double goal of (1) breeding individuals to release them in the wild while (2) preserving a captive stock with the maximum possible genetic diversity in the species [1]. These two types of strategies are complementary and have often been applied simultaneously in both plants and animals, particularly in critically endangered taxa [2,3]. Ex situ conservation programs often raise public interests, which facilitates large economic investments [4]. For instance, captive breeding programs have successfully been implemented for highly threatened animal species of different biological classes such as the Iberian lynx, *Lynx pardinus*; the black-footed ferret, *Mustela nigripes*; the whooping crane, *Grus americana*; the kakī, *Himantopus novaezelandiae;* and the Majorcan midwife toad, *Alytes muletensis* [5,6,7,8,9]. Although these programs have helped in the recovery of particular species, ex situ conservation strategies often face problems that may compromise their effectiveness. For instance, programs concerning highly threatened species are susceptible to inbreeding and the loss of genetic diversity, as the number of individuals used as founders is usually small and they can be genetically related [3]. In addition, captive breeding programs commonly face difficulties derived from the maintenance of wild species (e.g., physiological and behavioral maladaptations to captivity, parasites, and infectious diseases) [4]. In this context, necropsies of captive individuals are a very valuable tool. They provide useful information for better management of the captive stock, and help to determine the effect of environmental factors on the variation of physiological stress, as well as, consequently, on the mortality and population viability of threatened taxa [10].

**Figure 1:**
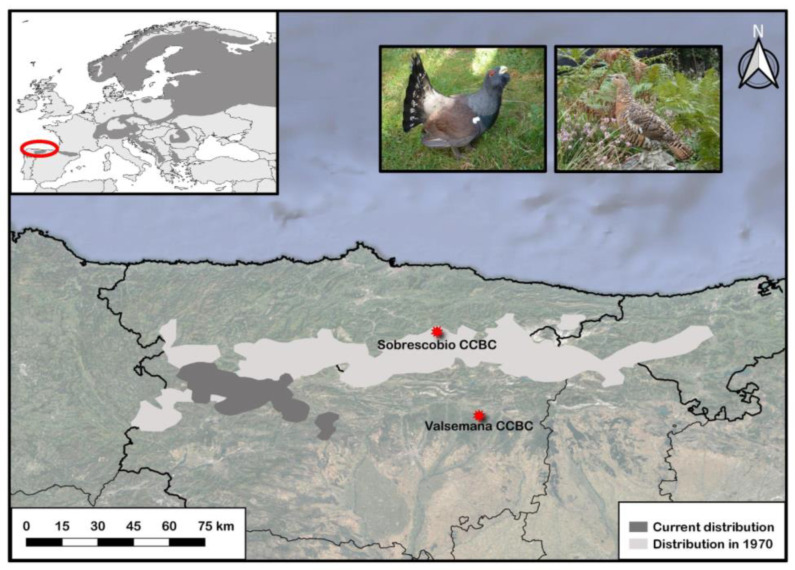
Map showing the Cantabrian capercaillie, *Tetrao urogallus cantabricus*, distribution range. Approximate distributions for 1970 and 2020, based on [17] are shown in light gray and dark gray, respectively. The locations of the Cantabrian Capercaillie Breeding Centers of Sobrescobio (Sobrescobio CCBC, Asturias province) and Valsemana (Valsemana CCBC, León province) are also shown. The **upper-left** figure represents the entire distribution range of the species in Europe. Images of male (**left**) and female (**right**) Cantabrian capercaillies can be seen at the top.

The western capercaillie *Tetrao urogallus* is a forest specialist grouse species, widely distributed across boreal and temperate regions of Eurasia [11] (Figure 1). Although abundant in Siberia and Scandinavia, the species is suffering a rapid decline in all its southernmost populations, which are usually located in highly anthropized and fragmented landscapes. Among the eight subspecies officially recognized [11], the Cantabrian capercaillie, *T. urogallus cantabricus*, is one of the most severely threatened, and the only one whose dominant habitat does not comprise coniferous forests, with the exception of some artificial plantations of Scotch pine, *Pinus sylvestris,* and Austrian pine, *P. nigra* [12,13]. Its current population range is located in the Cantabrian Mountains (northwestern Spain, Figure 1) and occupies primarily mature stands of deciduous forests of birch, *Betula pendula*; beech, *Fagus sylvatica*; and different oak species (*Quercus petraea, Q. orocantabrica Q. pyrenaica*) [12]. These singularities in habitat characteristics make the Cantabrian population unique in terms of feeding ecology and habitat use. For instance, as conifer needles (a key food in most capercaillie populations during winter) are rare in the area, Cantabrian capercaillies use more intensively understory food resources (bilberry stems, mosses, ferns, etc.) than those populations inhabiting boreal coniferous forests [14].

While the species is globally cataloged as “Least concern” by the International Union for Conservation of Nature (IUCN), Cantabrian capercaillies have recently been labeled as “Critically endangered” due to the rapid decline the subspecies is suffering [15]. Historically, the Cantabrian capercaillie occupied the majority of the Cantabrian Mountains, including its meridional extension, and areas of northern Portugal. Castroviejo et al. (1974) estimated that the Cantabrian capercaillie distribution occupied about 5300 km^2^ in the 1970s [16]. However, a recent study suggests that their current range has been restricted to an area smaller than 1000 km^2^, located in the western part of the mountain range (Figure 1), which suggests a reduction of 83% in their distribution in the last 50 years [17]. According to these authors, only around 200 capercaillies would remain in the Cantabrian Mountains nowadays [17]. Some causes seem to have acted synergistically in promoting the fast decline of this subspecies. For instance, factors such as habitat loss and fragmentation, land use changes, high densities of domestic ungulates, an increase in predators´ populations, and illegal hunting during the last century have been proposed as major threats to the conservation of Cantabrian capercaillies [14,18]. Being located at the southwestern edge of the species’ distribution and restricted to mountain areas, Cantabrian capercaillies are also likely to be among the populations more severely affected by climate change, which has already been associated with a decline in breeding success in the species [19]. In addition, the bilberry *Vaccinium myrtillus*, a key food and habitat resource for the species throughout its entire distribution range, is expected to constrain its current distribution to the Cantabrian Mountains due to the current warming scenario [20], which might decrease the availability of suitable habitat for capercaillies in the region. In order to stop the negative population trend, the Spanish Ministry of Environment created the National Strategy for the Conservation of the Cantabrian Capercaillie in 2004 [21]. This strategy included habitat management actions, and also the creation of a captive breeding program. Due to this, the first Cantabrian capercaillie breeding center opened in 2009 in Sobrescobio, Asturias province (“Sobrescobio CCBC” hereafter, Figure 1). In addition to this, and to avoid possible collapses of the captive stock, a second capercaillie breeding facility opened in Valsemana (León province) in 2022 (“Valsemana CCBC” in Figure 1). Since the start of the breeding program in 2009, a total of 74 capercaillies (including embryos) have died in the breeding facilities of Sobrescobio, from which the cause of death has been successfully identified in 29 individuals. Here, we use the data available from Sobrescobio CCBC to describe, for the first time, the main mortality causes of captive-bred Cantabrian capercaillies, and discuss how this new knowledge may provide relevant information for better maintenance of the species in captivity, as well as for the eventual adaptation to the wild for these animals. Additionally, the mortality causes of two free-ranging capercaillies are described.

## 2. Materials and Methods

### 2.1. Sobrescobio CCBC: Facilities and Management of Captive Capercaillies

Sobrescobio CCBC consists of six facilities of around 104 m^2^ each, which are subdivided into enclosures for males, females, and breeding, of 33.7, 25.2, and 45.1 m^2^, respectively. Enclosures are cleaned daily and captive animals are fed primarily with fodder, fruits, and vegetables, although pine needles, herbs, graminoids, ferns, and berries are also provided whenever available. Every year, in order to minimize inbreeding, males and females are selected to interbreed based on the individual genetic profiles obtained at the Laboratory of Genetics of the Veterinary Faculty at the Complutense University of Madrid. As a result of these mating events, female capercaillies have laid a total of 204 eggs in Sobrescobio CCBC since 2009, of which 56 hatched (27%), and 23 chicks survived (11%) [22].

### 2.2. Data Collection

Twenty-nine captive-bred Cantabrian capercaillies were necropsied between 2012 and 2022 in Sobrescobio CCBC. In total, 16 animals (5 males, 6 females and 5 undetermined) were chicks younger than 2 months (Table 1), whereas 13 (5 males and 8 females) were adults older than 7 months (Table 2). In addition, two free-ranging adult males found dead in 2004 and 2008 were also analyzed (Table 2). These two animals were found to be visiting peri-urban areas. They were trapped and reintroduced to the field, but they died shortly after; one of them had been bitten by a dog (showing severe traumatic lesions in coelomic cavity), and the other one was found dead.

We complied with the IUCN Policy Statement on Research Involving Species at Risk of Extinction and the Convention on the Trade in Endangered Species of Wild Fauna and Flora. We also complied with the national and regional legislation on sampling of Cantabrian capercaillie (Consejería de Fomento, Government of Principality of Asturias). Ethics approval was deemed unnecessary according to Spanish national regulations (Royal Decree 53/2013) [23].

### 2.3. Diagnostic Procedures

After necropsy, tissue samples (encephalon, spinal cord, heart, skeletal muscle, lungs, liver, kidneys, spleen, pancreas, gastrointestinal tract, genital tract, thymus, and Fabricio’s bursa) were obtained for evaluation using standard methods in histology. They were fixed in 10% neutral buffered formalin and subsequently submitted to the University of León or Servicio Regional de Investigación y Desarrollo Agroalimentario del Principado de Asturias (SERIDA) for further histopathological study. Tissue samples were processed routinely through graded alcohols prior to embedding in paraffin wax. Sections (4 μm) from each block were mounted on separate glass microscope slides and stained with hematoxylin and eosin, Gram, Ziehl-Neelsen, von Kossa, Mallory Azan, Masson´s trichrome, and periodic acid-Schiff (PAS).

Fresh samples were also collected in order to perform bacteriological and molecular studies. Bacteriological studies were performed using three commercial media following the manufacturers’ instructions: Columbia agar with 5% sheep blood for aerobic bacteria isolation (Biomérieux, Madrid, Spain), Schaedler for anaerobic bacteria isolation (Biomérieux, Madrid, Spain), and Sabouraud Chlora-Genta for fungal isolation (Thermo Scientific, Waltham, MA, USA). Culture plates for aerobic or anaerobic bacterial isolation were subsequently incubated at 37 °C for 24–48 h, in aerobiosis or anaerobiosis, respectively. Afterwards, Gram staining was performed, and aerobic bacteria were biochemically identified, either using VITEK^®^2 GN cards (Biomérieux, Madrid, Spain) for Gram-negative microorganisms, or VITEK^®^2 GP cards (Biomérieux, Madrid, Spain) for Gram-positive microorganisms, in VITEK^®^2 Compact equipment (Biomérieux, Madrid, Spain). Similarly, anaerobic bacteria were identified using VITEK^®^2 ANC cards (Biomérieux, Madrid, Spain) in the same equipment. Fungal culture plates were incubated at room temperature for 7 days in aerobiosis in a humid chamber. In case of fungal growth, a specific staining with lactophenol blue was performed and were then morphologically identified using an optical microscope.

The conventional polymerase chain reaction (PCR) technique for the identification of *Aspergillus fumigatus* was performed in lung and air sac samples from adult capercaillie 3, following a published protocol [24]. Briefly, the PCR was performed with 5 µM of each primer (5′-GCCCGCCGTTTCGAC-3′ and 5′-CCGTTGTTGAAAGTTTTAACTGATTAC-3′) for the specific amplification of *A. fumigatus* ITS1 region, using the DNA AmpliTools Multiplex Master Mix (Biotools, Madrid, Spain) in a GenAmp PCR System 9700 (Thermofisher Scientific, Barcelona, Spain).

Additionally, coprological assays were routinely performed in the breeding center using fresh fecal samples collected from the cages since 2015. Data from years 2022 and 2023 were available in this study. Two grams of each fecal sample (*n* = 41) was diluted in 27 mL of supersaturated solution of sugar and salt (1 L of distilled water + 400 g of salt + 500 g of sugar) and microscopically studied by flotation analysis at 100× magnification using a MacMaster egg counter slide for quantification.

Virological analyses were not conducted in this study, since we found neither pathological nor clinical features indicative of viral disease.

Data on necropsy findings and laboratory results were considered in making conclusions about the causes of death.

## 3. Results

### 3.1. Classification of Causes of Death

The confirmed causes of death and relevant pathological findings in the 31 capercaillies studied are shown in Table 1 (young animals less than 2 months old) and Table 2 (adult animals more than 7 months old). In the 29 captive capercaillies, causes of death were classified as infectious (18/29, 62.07%), mainly due to *Escherichia coli, Clostridium perfringens,* or *A. fumigatus* infection, or non-infectious (11/29, 37.93%), due to stress-related processes (i.e., rupture of the heart apex and cardiomyopathy or neurogenic shock) (8/29, 27.59%), duodenal obstruction and coelomitis (1/29, 3.45%), perforation of the proventriculus and heart with a briar branch (1/29, 3.45%), or euthanasia due to a valgus leg deformity that prevented proper animal welfare (1/29, 3.45%). When age was considered, young animals mainly died due to infectious diseases (14/16, 87.5%) (Table 1), and adult animals mainly due to stress-related causes (7/13, 53.85%), followed by infectious bacterial diseases (3/13, 23.08%) (Table 2). No relevant differences were found between sexes. Coprological analysis morphologically identified 29.27% (12/41) of fecal samples collected in cages positive to protozoan coccidian parasite *Eimeria* spp., and 7.32% (3/41) positive to the nematodes *Capillaria* spp. (*n* = 1) and *Trichontrongylus* spp. (*n* = 2); however, both coccidia and nematodes were found in very small quantities, and they were not associated with clinical signs of disease, such as diarrhea. The two free-ranging capercaillies died due to exertional myopathy (degenerative lesions). Main causes of death are described in Section 3.2.

### 3.2. Description of Causes of Death

#### 3.2.1. Infectious Diseases

##### Bacterial Diseases

**Figure 2:**
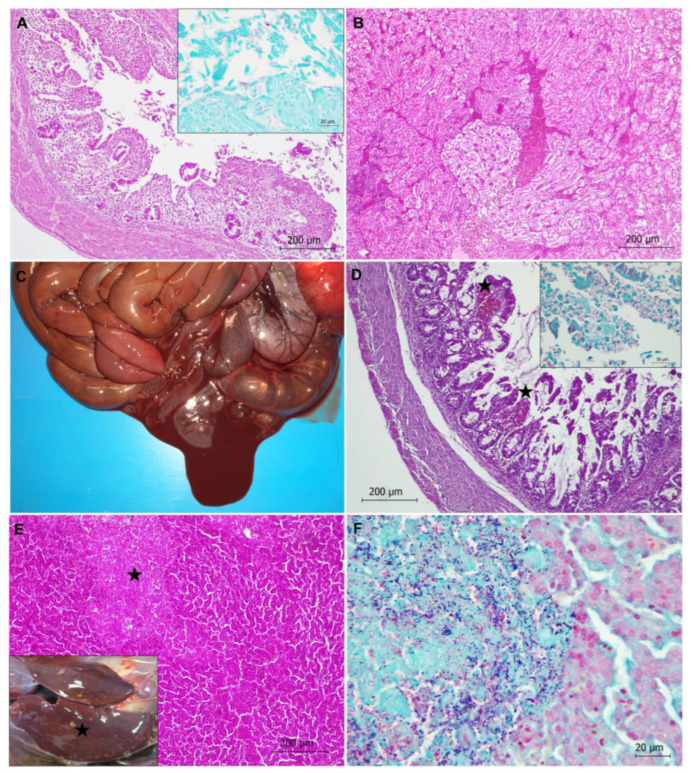
Colibacillosis (**A**,**B**) and clostridiosis (**C**–**F**) in Cantabrian capercaillies. (**A**) Typhlitis due to *Escherichia coli* infection is observed, characterized by epithelial cell damage of the mucosa and inflammatory infiltrate. Hematoxylin-eosin stain. Inset: Gram-negative bacilli compatible with *E. coli* are present on the apical surfaces of mucosal epithelial cells. Gram stain. (**B**) Congestion and hemorrhage in kidney of an animal with colibacillosis. Hematoxylin-eosin stain. (**C**) Hemorrhagic enteritis is observed in a capercaillie with *Clostridium perfringens* infection. (**D**) Histological features in the intestine of a capercaillie with clostridiosis. Necrotic typhlitis is observed; stars indicate hemorrhages. Destruction of intestinal mucosa and network of fibrin are also detected. Hematoxylin-eosin stain. Inset: Presence of numerous Gram-positive bacilli adhered to the mucosal epithelium are shown. Gram stain. (**E**) Necrotic hepatitis with a focus of necrosis (star) in the liver parenchyma is observed. Hematoxylin-eosin stain. Inset: Multiple whitish-yellowish foci of necrosis (star) are observed in the liver. (**F**) Numerous Gram-positive bacilli compatible with *Clostridium* spp. are located in the focus of necrosis observed in (**E**). Gram stain.

In total, 17 captive capercaillies (17/29, 58.62%) died due to bacterial disease, septicemia and/or endotoxic shock, characterized by congestion, edema, hemorrhages, and microthrombi in the vessels of several organs, such as the liver, kidneys, spleen, lungs, and brain (Figure 2). In 14 animals, the etiological agent could be identified by microbiology. The involved bacteria were *E. coli* in 10 animals (10/29, 34.48%), *C. perfringens* in 2 animals (2/29, 6.90%), *Enterococcus* spp. in 1 animal (1/29, 3.45%) and *E. coli*, *C. perfringens*, and *E. gallinarum* in another animal (1/29, 3.45%). In total, 14 out of the 17 animals showing bacterial disease were chicks less than 2 months old. None of those animals showed parasitic infection due to either coccidia or nematodes in intestinal samples.

In total, 6 out of the 11 animals with *E. coli* infection showed acute enteritis, more severe in caeca (typhlitis), with or without impaction. Enteritis was characterized by an inflammatory infiltrate consisting of lymphocytes and macrophages and epithelial cell damage of the mucosa (Figure 2), with microerosions of the mucosa surface. Gram-negative bacilli compatible with *E. coli* were found on the apical surface of mucosa. The remaining animals with *E. coli* infection showed septicemia or sacculitis.

One capercaillie showed necrotic hemorrhagic enteritis due to *C. perfringens* bacterium (Figure 2). Diffuse necrosis of the mucosa was observed. Gram-positive bacilli colonization of the tip of the villi with associated hemorrhages and networks of fibrin in the lumen of the intestine (mainly in caeca) were also noticed (Figure 2).

Three capercaillies showed necrotic hepatitis (Figure 2). Well-demarcated multi-focal necrosis was observed in the liver parenchyma without an associated inflammatory infiltrate. Gram staining revealed clumps of bacilli morphologically compatible with *Clostridium* spp. in the pale, amorphous necrotic parenchyma (Figure 2). In one case, *C. perfringens* was identified via microbiology. One of these animals showed neurological clinical signs compatible with hepatic encephalopathy.

Purulent myocarditis characterized by hemorrhages, an inflammatory infiltrate of neutrophils, and lymphocytes between myocardial fibers, as well as the presence of a moderate number of Gram-positive coccobacteria, was observed in an 8-year-old female capercaillie. The causative bacterium could not be identified.

##### Generalized Aspergillosis

**Figure 3:**
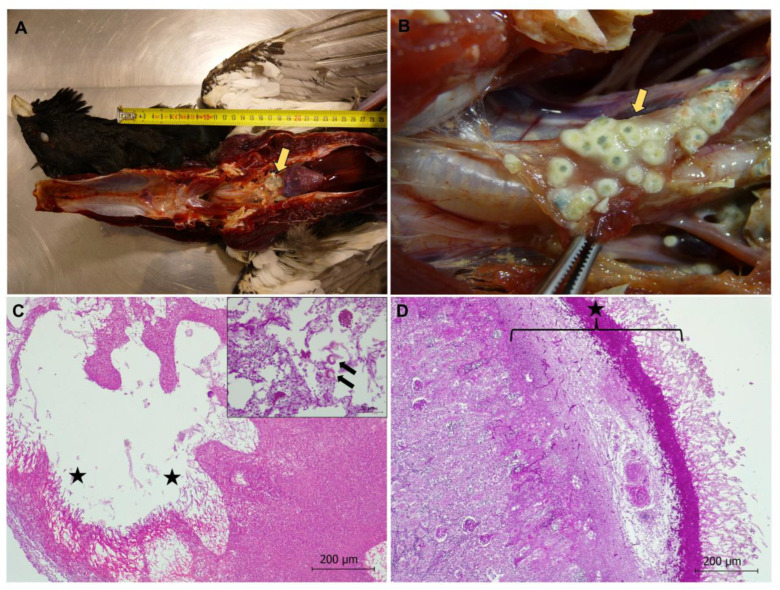
Aspergillosis in a Cantabrian capercaillie. (**A**) Gross lesion (arrow) due to *Aspergillus fumigatus* in a capercaillie. (**B**) Numerous nodules (greenish-white granulomas, arrow) in air sacs of a capercaillie with acute aspergillosis are observed. (**C**) Lung. Severe pneumonia is observed, with a parabronchus containing numerous, dichotomously branched, septate fungal hyphae (stars), disclosed using PAS staining. Inset: Conidiophores (arrows) are seen in this severe pulmonary infection. PAS stain. (**D**) Kidney. Granulomatous nephritis with numerous hyphae and few conidiophores in the renal cortex (star) are observed. PAS stain.

Generalized aspergillosis was observed in adult capercaillie number 3. Gross lesions consisted of several greenish-white fungal granulomas (2–5 mm in diameter, approximately) located in air sacs, lungs, kidneys, and in the surface of the vertebral column (Figure 3). A vertebral column granuloma was likely compressing the spinal cord, suppressing tail mobility. Histologically, granulomatous airsacculitis, pneumonia, and nephritis were observed. Granulomas contained numerous, dichotomously branched, septate fungal hyphae radiating from the center of the lesions, which were revealed using PAS staining (Figure 3). An inflammatory infiltrate consisting of macrophages, a small number of lymphocytes, and fibroblasts was observed within the granulomas. PCR identified *A. fumigatus* as the etiological agent.

#### 3.2.2. Non-Infectious Diseases

##### Neurogenic Shock

**Figure 4:**
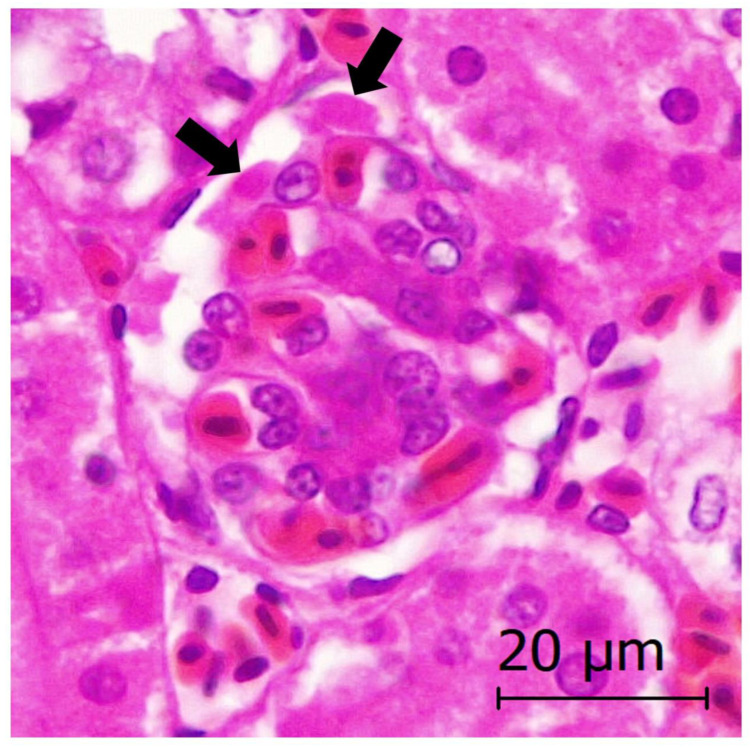
Stress-related neurogenic shock in a Cantabrian capercaillie. Disseminated intravascular coagulation-DIC (i.e., microthrombi, arrows) in a renal glomerulus is observed.

Four adult animals showed neurogenic shock, possibly triggered by stressful situations (e.g., two animals died after management). Generalized vascular lesions were observed and consisted of congestion, edema, hemorrhages, and disseminated intravascular coagulation-DIC (i.e., blood clots or microthrombi formation throughout the body, blocking small blood vessels) in several organs such as the brain, lungs, liver, and kidneys (Figure 4).

##### Rupture of the Heart Apex and Cardiomyopathy

**Figure 5:**
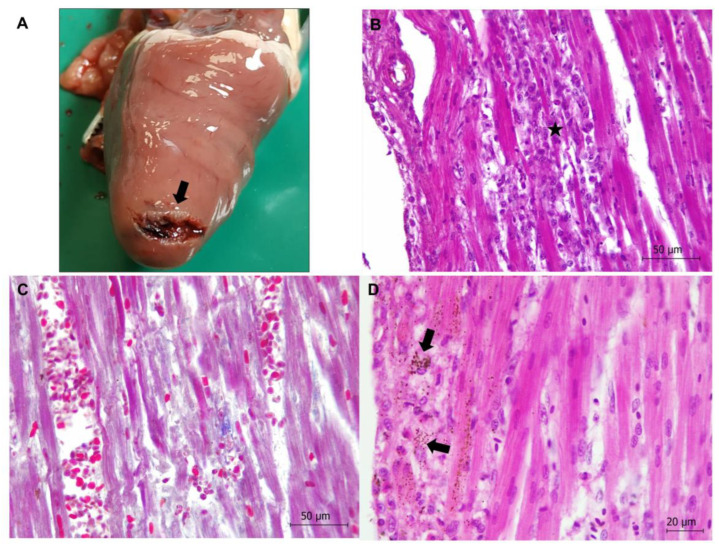
Rupture of the heart apex and cardiomyopathy in a Cantabrian capercaillie. (**A**) Rupture of the heart apex with associated hemorrhage is observed (arrow). (**B**) Fragmentation of myocardial fibers, as well as prominent inflammatory infiltrate mainly made up of macrophages (star), can be observed. Hematoxylin-eosin stain. (**C**) Myodegeneration is highlighted using Mallory Azan staining. Only the unaffected fibers are stained in red. (**D**) Calcification (mineralization) (in black, arrows), hyalinization, necrosis of muscle fibers, and an infiltrate of macrophages are observed. Von Kossa stain.

Three captive capercaillies, one young and two adults, suddenly died due to a rupture of the heart apex (Figure 5). Hemopericardium was the main gross lesion observed. Microscopically, subacute cardiomyopathy was also diagnosed, comprising myodegeneration with fragmentation of myocardial fibers, flocculation of the necrotic sarcoplasm, mineralization with marked basophilic granularity, and a prominent infiltrate composed mainly of macrophages (Figure 5). A fourth capercaillie showed cardiomyopathy without rupture of the heart apex.

##### Valgus Leg Deformity

**Figure 6:**
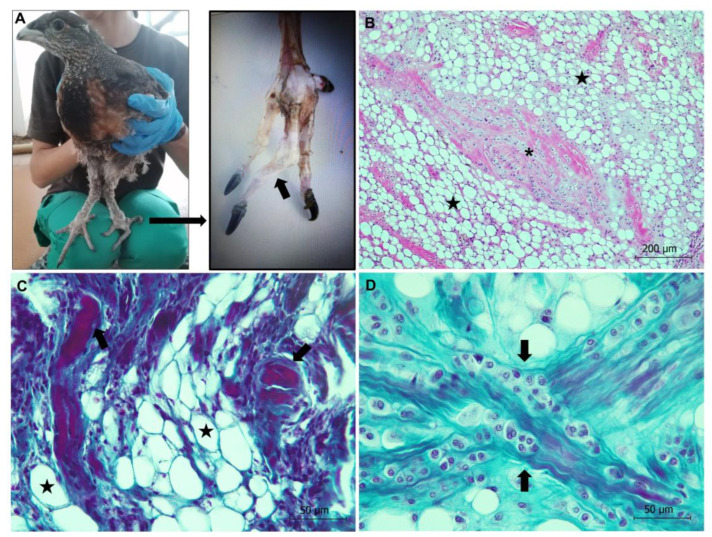
Valgus leg deformity in a Cantabrian capercaillie. (**A**) The left leg is bent outwards at the hock joint (left image). The animal also shows lateral tilting of the distal tibiotarsal condyles (arrow, right image). (**B**) Myotonic dystrophy (stars) and fibrous cartilaginous metaplasia (asterisk) are observed in the region of the tendon and skeletal muscle associated with the femoral head. Hematoxylin-eosin stain. (**C**) Degeneration of muscle fibers that are atrophied (in red, arrows) and replaced by adipose tissue (stars) is observed using Masson´s trichrome stain. (**D**) Detail of the fibrous cartilage metaplasia, where chondrocytes are organized forming parallel rows between the collagen bundles (arrows). Masson´s trichrome stain.

Valgus leg deformity was diagnosed in a 2-month-old male (animal 16). The deformity caused the left leg to bend outwards at the hock joint, as well as tilting of the distal tibiotarsal condyles (Figure 6). The animal showed clinical signs of stiffness. Histologically, myotonic dystrophy and fibrous cartilaginous metaplasia were observed in the region of the tendon and skeletal muscles associated with the femoral head (i.e., musculus tibialis internus and musculus quadratus) (Figure 6). Myotonic dystrophy consisted of degeneration of muscle fibers that were atrophied and replaced by adipocytes. There was no gross or histological evidence of infection.

##### Duodenal Obstruction and Proventriculus Perforation

**Figure 7:**
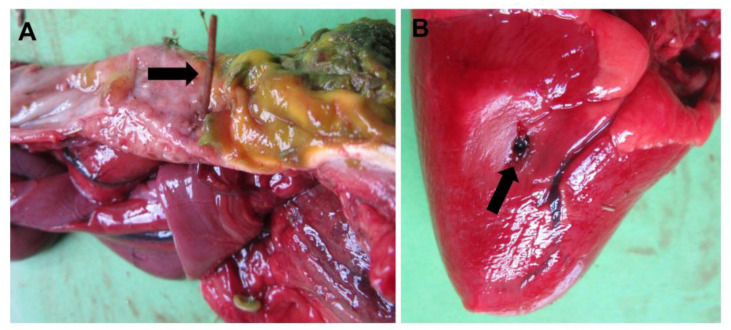
Proventriculus perforation with a briar branch (**A**, arrow) and subsequent right heart ventricle perforation (**B**, arrow) in a capercaillie.

Two adult capercaillies died due to diet-related causes. One of them showed a duodenal obstruction after eating a hazelnut that subsequently caused necrosis of the intestinal wall and coelomitis. The other one showed perforation of the proventriculus with a briar branch, and afterwards perforation of the pericardium and right ventricle of the heart, which finally caused the death of the animal (Figure 7).

##### Exertional (Degenerative) Myopathy in Two Free-Ranging Capercaillies

**Figure 8:**
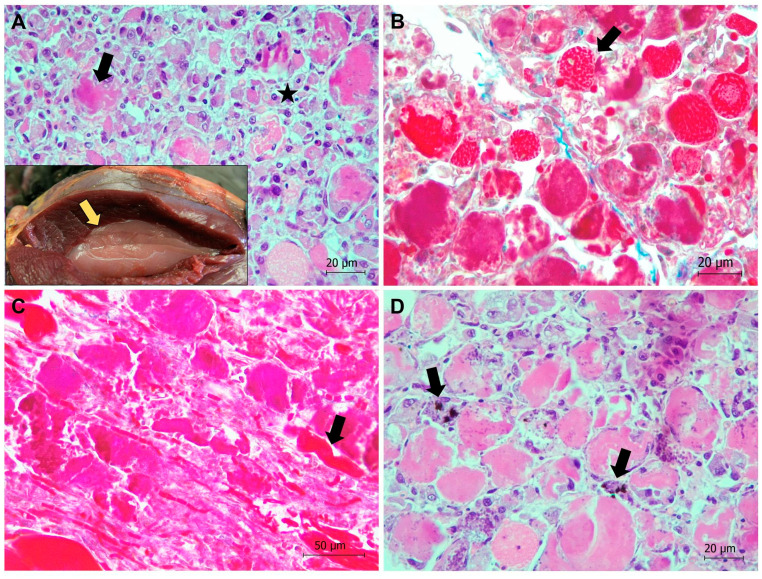
Severe exertional myopathy in a Cantabrian capercaillie in the supracoracoideus muscle. (**A**) Extensive segmental hyaline degeneration in the supracoracoideus muscle. Coagulative necrosis, areas of myofibrillar lysis (black arrow), as well as intensive inflammatory infiltrate mainly consisting of lymphocytes and macrophages (star) are observed. Hematoxylin-eosin stain. Inset: Gross appearance of the supracoracoideus muscle, which appears pale and dry (yellow arrow). (**B**) Hypercontracted, necrotic, and lysed myofibers are highlighted using Mallory Azan stain (arrow). (**C**) Segmental degeneration and inflammatory infiltrate in a longitudinal section of supracoracoideus muscle. Surviving myofibers can be seen in red (arrow). Mallory Azan stain. (**D**) Calcification (mineralization) (in black, arrows), hyalinization, and necrosis of muscle fibers are observed. Von Kossa stain.

In the two free-ranging capercaillies, exertional myopathy was diagnosed in the pectoralis and/or supracoracoideus muscles, which showed gross lesions consisting of dry and pale muscle (also called “wooden breast”) (Figure 8). Microscopically, severe segmental degeneration of muscle was observed, consisting of hypercontracted fibers, extensive Zenker´s hyaline degeneration, and coagulative necrosis of myofibers, more severe in the capercaillie identified as animal 1 (Figure 8). This animal also showed an intensive inflammatory infiltrate mainly consisting of lymphocytes and macrophages, as well as mineralization in the affected muscles (Figure 8). Additionally observed in that animal, necrotic myofibers presented surviving satellite cells, invading macrophages, and elongating myoblasts, all indicative of events of regeneration (Figure 8). Mineralization was confirmed by von Kossa´s histological technique, which stains carbonates and phosphates in black (Figure 8).

## 4. Discussion

Here, we describe, for the first time, the main mortality causes of captive-bred Cantabrian capercaillies, members of a subspecies of western capercaillie that is critically endangered. This work reveals that infectious bacterial diseases represented the most frequent cause of death in captive capercaillies, mainly affecting newborn and young animals (< 2 months old, 87.5%), whilst stress-related processes, such as neurogenic shock or cardiomyopathy, caused the death of most animals older than 7 months old (53.85%), followed by infectious bacterial diseases (23.08%). Our results are in line with those reported by previous studies analyzing the mortality causes of captive tetraonids. For instance, infectious diseases, mainly in gastrointestinal processes, were the most common mortality causes of captive Attwater´s prairie chickens, *Tympanuchus cupido*, affecting both young and adult animals [25]. Similarly, capture myopathy and handling injuries are commonly reported in other captive bird species [26,27]. On the other hand, we believe that the duodenal obstruction with a hazelnut and proventriculus and heart perforation with a briar branch observed in this study represent isolated cases, although they suggest the susceptibility of western capercaillies to some food items present in their diet. Viral diseases, often diagnosed in captive psittacine birds [28], were not present in this study based on pathological studies.

Among bacterial diseases, the proportion of animals dead due to colibacillosis was the highest, followed by clostridiosis. Both are important infectious diseases in chicks, and affect poultry worldwide [29,30,31], with several cases of *C. perfringens* infection causing necrotizing enteritis or necrotic hepatitis reported in captive capercaillies [32]. The etiological agents—*E. coli* and *C. perfringens*—use several virulence strategies to achieve tissue invasion such as metabolic enzymes, adhesion molecules, or tissue-degrading toxins [31,33]. In this regard, *E. coli* typing (e.g., detection of enterotoxigenic *E. coli*-ETEC, enteroinvasive *E. coli*-EIEC, enteropathogenic *E. coli*-EPEC, or enterohemorrhagic *E. coli*-EHEC), should be considered in future studies in order to better understand and prevent this bacterial infection [32]. Gut microbiota plays a key role in maintaining the physiological structure and function of the intestine [34]. In bird and mammal species, *E. coli* and *C. perfringens* are usually part of the normal gut microbiota, but they can proliferate under several circumstances that may favor dysbiosis (i.e., microbiota imbalance). Known predisposing factors for dysbiosis include stress, unclean environment, weak immune system, diet changes, high dietary protein, or soluble non-starch polysaccharides, wheat- or maize-based diets (high in polysaccharides), antibiotic administration, or primary coccidiosis [34,35,36,37]. In our study, coccidia were identified in 29% of the fecal samples collected from cages, but always at very low levels, not implying clinically related signs in capercaillies. Moreover, none of the capercaillies dead due to bacterial disease showed coccidia infection in the intestine; therefore, we excluded coccidiosis as a predisposing factor. Interestingly, *E. coli* and *C. perfringens* are species commonly found in the fecal samples of captive capercaillies [38,39], but not in free-living ones [39], a fact which highlights the relevance of maintaining a proper gut microbiota balance in captive animals. This might be achieved by mimicking the natural diet of wild animals. For instance, we believe that a diet based on natural food items such as (1) beech buds, ferns, and bilberry leaves during spring; (2) bilberry leaves and fruits, as well as ferns during summer and autumn; and (3) holly leaves, bilberry shoots, and ferns during winter [14] might enhance the gut microbiota of captive Cantabrian capercaillies. In this regard, the bacterial microbiota in the caeca of capercaillies usually differs between wild and captive animals [40]. As an example, while certain lineages of Clostridiales, Synergistetes, and Actinobacteria are most prevalent in wild birds, they are strongly reduced in individuals raised in captivity. Of additional interest is the complete absence of Megasphaera and Synergistes species in captive capercaillies, which in turn usually results in a large abundance of Gammaproteobacteria, bacteria closely related to Anaerobiospirillum members and commonly connected with intestinal dysfunction [40].

In recent decades, antibiotics were used in the poultry industry to prevent or control colibacillosis and clostridiosis in animals, but due to the increasing drug residues and drug-resistant bacteria issues, alternatives are being applied to protect chickens, such as (1) the use of probiotics and prebiotics, (2) live yeast and mannan-oligosaccharide supplementation in the diet to improve microbial community structure, or (3) vaccination strategies [41,42,43]. Similar strategies might be used in captive capercaillies to control or attenuate those diseases, with the aim of protecting the health status of the animals. Additionally, the lack of natural antibacterial substances in the feed of captive capercaillies may be one of the reasons for the occurrence of *E. coli*, other Enterobacteriaceae, and *C. perfringens* in those animals [44], a possibility which additionally supports that captive capercaillies, especially young animals to be released in the wild, must be fed with natural fodder plants [44]. For instance, insect protein must represent an important fraction of the diet of captive chicks (70–80% of the total energy intake during the first 4 weeks) in order to ensure an optimal growth performance [45].

One animal showed generalized aspergillosis. *Aspergillus fumigatus*, a filamentous fungus first described in the lungs of a great bustard, *Otis tarda*, by Gerog Fresenius in 1863 [46], is considered a major respiratory pathogen in birds. Infection by *Aspergillus* spp. has been reported in almost all domesticated avian species and production types [47,48]. While acute aspergillosis generally occurs in young birds, and is associated with high morbidity and mortality, the chronic form is sporadic and causes lesser mortality, generally affecting older birds with compromised immune systems (e.g., poultry breeders). Poor husbandry conditions, such as high humidity, poor ventilation and sanitation, warm temperature conditions, and long-term storage of feed, can promote the rapid growth of hyphae and efficient asexual multiplication resulting in the copious production of spores in the air [48], which are subsequently dispersed and inhaled by the birds [47]. In addition, factors that compromise the bird’s immunity, or an inadequate diet resulting in hypovitaminosis A, can also predispose the animal to mycosis [49]. Conidiophores, such as those found in the capercaillie dead due to aspergillosis in this study, are generally associated with severe pulmonary infections, particularly when fungal growth takes place in the airspace. We believe that protective immunity following vaccination may help to treat and prevent avian aspergillosis, although we recall that vaccination strategies may not be applicable for immunosuppressed animals requiring passive immunization with immunoglobulins [50].

Eight captive and two free-ranging capercaillies died after severe stress, due to cardiomyopathy (with subsequent rupture of the heart apex), neurogenic shock, or exertional myopathy (see Table 1 and Table 2). Avian myopathy has been correlated with stress in chickens, and also in capercaillies [51,52], with a pathway that leads to hypoxia and oxidative stress with alteration of mitochondrial homeostasis, release of Ca^2+^ following the collapse of the sarcoplasmic reticulum, activation of intracellular proteases, and eventual muscle fiber breakdown [53]. Dietary Selenium and Vitamin E (Se/Vit E) deficiency may cause nutritional muscular dystrophy or myopathy of chicks in both cardiac and skeletal muscles, associated with oxidative stress damage, which promotes DNA fragmentation, chromatin condensation, and finally apoptosis. We do not know if capercaillies showing myopathy or cardiomyopathy in this study suffered from Se/Vit E deficiency, which might have favored the onset of the disease, but the addition of 0.3 mg Se/kg to the diet can prevent myopathy likely exerted via control of oxidative stress [54,55]. In addition, post mortem Se/Vit E detection might be helpful in future necropsies of capercaillies in order to determine the mineral and vitamin levels in affected animals. The pathogenesis of the rupture of the heart apex in three capercaillies is unknown. However, we observed remarkable similarities in morphology and location of the lesions with those previously described in three capercaillies [51]. This could indicate a great anatomic predisposition to this lesion in the species. We suggest that the cardiomyopathy observed in the capercaillies might have favored the rupture of the heart apex. Similar conclusions were obtained by [51], suggesting a species predisposition to this lesion. On the other hand, neurogenic shock is produced due to severe stress originating in the autonomic nervous system [56]. High stress levels cause adrenalin liberation, which leads to (1) peripheral vasodilatation, (2) a critical loss in the effective circulating blood volume due to an increased vascular permeability, and (3) a systemic hypoperfusion state, triggering cellular hypoxia and dysfunction, and finally the death of the animal, usually between 1 and 6 h after the stressful situation [56].

One captive capercaillie showed valgus leg deformity, also called valgus twisted legs. This lesion seems to be rare in western capercaillies, although a similar case was previously reported in a free-ranging individual from the Pyrenean population [57]. Valgus leg deformity was first described in broilers [58], referring to lateral angulation of tibiotarsal articulation. This deformity is usually seen in broiler chickens from about 2 weeks of age onwards, with one leg or both affected, and more often in males than in females [59]. Many studies have concluded that valgus leg deformity is heritable [60], although this remains unclear. In addition, low calcium or zinc in the diet, irregular vascular supply of the growth plate, delayed cortical bone differentiation, and lack of exercise have been suggested as predisposing factors [61,62,63,64]. Furthermore, a restriction of metabolizable energy intake during the first 14 days after hatching halved the incidence of leg deformities in broilers [62]. In any case, the disease is likely to be an isolated case in capercaillie. This capercaillie also showed metaplasia of the fibrous cartilage, which is usually associated with chronic injury as an adaptative response of the tissue involved [65].

## 5. Conclusions

In summary, and considering our limited sample size, bacterial diseases (mainly colibacillosis and clostridiosis) appear to be the most prevalent cause of death in young captive capercaillies, thus limiting survival in captivity. In adult capercaillies, the main cause of death is related to stress, although infectious diseases are also relevant. This study provides valuable information that may help to reduce mortality and increase the utility and effectiveness of captive breeding centers in the conservation strategy of the species. Wild and captive capercaillies differ in many morphological and physiological features that must be considered during breeding and management, such as the cytochrome C oxidase activity and glycogen content of the muscles, the size of the heart, the weight of the gizzards, and the length of the small intestine and caeca, which may impact effective decomposition and absorption activities [40,66]. On the other hand, the two necropsies of free-ranging capercaillies showed the presence of exertional myopathy, suggesting a similar predisposition to stress-related mortality causes to those reported in captive conditions. In this regard, more resources must be invested (e.g., radio tracking of reintroduced animals) in order to gain a deeper knowledge of the main threats compromising the survival of wild Cantabrian capercaillies and, consequently, the recovery of the entire population.

## Figures and Tables

**Table 1:** Available data, cause of death, and classification of death in young captive Cantabrian capercaillies (*Tetrao urogallus cantabricus*) necropsied in Spain.

ID	Date	Age	Sex	Cause of Death	Microbiology	Classification of Death
1	4 July 2015	6 days	N.d.	Acute enteritis/septicemia	Culture: *Enterococcus* spp. (intestine, liver)	I
2	29 April 2016	5 days	N.d.	Septicemia	Culture: *Escherichia coli* (intestine, liver)	I
3	31 July 2017	47 days	Female	Septicemia	Culture: *Escherichia coli* (intestine, liver, kidney)	I
4	25 June 2019	4 days	N.d.	Sacculitis	Culture: *Escherichia coli* (sacculum)	I
5	25 June 2019	3 days	N.d.	Septicemia	Culture: *Escherichia coli* (intestine, liver)	I
6	25 June 2019	3 days	Male	Acute typhlitis	Culture: *Escherichia coli* (intestine)	I
7	12 July 2019	8 days	Male	Acute enteritis	Culture: *Escherichia coli* (intestine)	I
8	3 July 2020	8 days	Male	Acute enteritis/septicemia	Culture: *Escherichia coli* (intestine, liver)	I
9	3 July 2020	8 days	N.d.	Septicemia	Culture: *Escherichia coli* (fecal content, liver)	I
10	16 July 2020	15 days	Male	Acute enteritis/septicemia	Culture: *Escherichia coli* (intestine, liver)	I
11	2 July 2021	15 days	Male	Acute typhlitis	Culture: *Escherichia coli, Enterococcus gallinarum*, *Clostridium perfringens* (intestine)	I
12	12 July 2022	6 days	Female	Necrotic hemorrhagic enteritis/septicemia	Culture: *Clostridium perfringens* (intestine, liver)	I
13	20 July 2022	26 days	Female	Acute enteritis/septicemia	Culture: *Escherichia coli* (intestine, liver, heart, brain)	I
14	21 July 2022	6 days	Female	Necrotic hepatitis	-	I
15	15 September 2022	55 days	Female	Heart apex rupture/cardiomyopathy	-	NI
16	10 November 2022	2 months	Male	Valgus leg deformity	-	NI-euthanasia

ID: Animal identification; N.d.: not determined; I: infectious; NI: non-infectious. Organs within parentheses in microbiology column indicate those organs from which positive results were obtained.

**Table 2:** Available data, cause of death and classification of death in adult Cantabrian capercaillies (*Tetrao urogallus cantabricus*) necropsied in Spain.

ID	Date	Age	Sex	Cause of Death	Microbiology	Classification of Death
1	14 April 2004	3 years	Male	Exertional myopathy	-	NI
2	12 July 2008	Adult	Male	Predation-Exertional myopathy	-	NI
3	19 June 2012	1 year	Male	Aspergillosis	PCR: *Aspergillus fumigatus* (lungs, air sacs)	I
4	12 November 2013	2 years	Male	Duodenal obstruction with a hazelnut and coelomitis	-	NI
5	26 January 2017	1.5 years	Female	Neurogenic shock (stress)	-	NI
6	3 February 2017	7 months	Female	Cardiomyopathy	-	NI
7	3 February 2017	7 months	Female	Heart apex rupture/cardiomyopathy	-	NI
8	26 January 2018	2.5 years	Female	Neurogenic shock (stress)	-	NI
9	27 June 2018	1 year	Male	Neurogenic shock (stress)	-	NI
10	15 March 2019	3.5 years	Female	Perforation of the proventriculus and heart with a briar branch	-	NI
11	1 March 2020	8 years	Female	Heart apex rupture/cardiomyopathy	-	NI
12	20 June 2022	7 years	Male	Necrotic hepatitis	Culture: *Clostridium perfringens* (intestine, liver, heart)	I
13	6 July 2022	10 years	Male	Necrotic hepatitis	-	I
14	9 November 2022	5 years	Female	Neurogenic shock (stress)	-	NI
15	17 November 2022	8 years	Female	Purulent myocarditis	-	I

ID: Animal identification; I: infectious; NI: non-infectious; PCR: polymerase chain reaction. Gray shade indicates free-ranging capercaillies; the remaining capercaillies were captive. Organs within parentheses in microbiology column indicate those organs from which positive results were obtained.

## Data Availability

The data that support the findings of this study are available from the corresponding author upon reasonable request.
